# Robust Transition Metal Contacts for Aligned Carbon Nanotubes

**DOI:** 10.3390/nano15100736

**Published:** 2025-05-14

**Authors:** Gang Huang, Junhong Wu, Haiou Li, Honggang Liu

**Affiliations:** 1 Guangxi Key Laboratory of Precision Navigation Technology and Application, Guilin University of Electronic Technology, Guilin 541004, China; 2 School of Integrated Circuits, Beijing University of Posts and Telecommunications, Beijing 100876, China; 3 Center for Carbon-Based Electronics and School of Electronics, Peking University, Beijing 100871, China; 4 Chongqing Institute of Carbon-Based Integrated Circuits, Peking University, Chongqing 401332, China

**Keywords:** transition metal carbides, contact resistance, aligned carbon nanotube, rapid thermal annealing, Schottky barrier height

## Abstract

Aligned carbon nanotubes (A-CNTs) are emerging as one of the most promising materials for next-generation nanoelectronics. However, achieving reliable ohmic contacts between A-CNTs and metals remains a critical challenge. In this study, we employ rapid thermal annealing (RTA) to facilitate the formation of transition metal carbides at the metal–CNT interface, significantly reducing contact resistance and enhancing stability. Using the transmission line method (TLM), we demonstrate that RTA reduces the contact resistance at the Ti/A-CNT interface from 112.26 kΩ·μm to 1.57 kΩ·μm and at the Ni/A-CNT interface from 81.72 kΩ·μm to 1.17 kΩ·μm, representing a reduction of over an order of magnitude. Moreover, the Schottky barrier heights (SBHs) for both the Ti/A-CNT and Ni/A-CNT interfaces decreases by approximately 50% after annealing. A comparative analysis with Pd/A-CNT contacts shows that Ti and Ni contacts exhibit superior reliability under harsh conditions. This work provides a viable solution for improving the electrical performance and reliability of CNT-based devices, offering a pathway toward the development of future CMOS technologies.

## 1. Introduction

In the post-silicon era, carbon nanotubes (CNTs) are emerging as promising candidates for the next generation of nanoelectronic devices and integrated circuits (ICs) due to their exceptional electrical properties, high carrier mobility, atomic-scale thickness, scalability, and compatibility with complementary metal–oxide–semiconductor (CMOS) technology [1,2,3,4,5]. Among these, aligned carbon nanotubes (A-CNTs) are particularly suited as transistor channel materials for future CMOS applications [6]. However, one of the key challenges in creating high-performance CNT-based devices is achieving reliable, low-resistance ohmic contacts between CNTs and metal contacts [7]. Properly engineered ohmic contacts reduce on-state resistance ($R_{\mathrm{on}}$), thereby ensuring efficient electron transmission, and greatly improving the overall yield and reliability of CNT-based integrated circuits [8].

Recent advancements in high-performance field-effect transistors (FETs) utilizing A-CNT films as channel materials have demonstrated their viability for future CMOS applications. Liu et al. demonstrated A-CNT-based CMOS FETs with symmetric performance for both PMOS and NMOS, achieving hole and electron mobilities of 325 cm^2^/V·s and 241 cm^2^/V·s, respectively [9]. Chen et al. introduced various logic gates, shifters, and D-latch circuits with rail-to-rail output. Notably, a 4-bit adder composed of 140 p-type CNT FETs exhibited higher packing density and lower supply voltage than other CNT-based integrated circuits, thus demonstrating the feasibility of medium-scale CNT-based ICs [10].

The stability of the Ohmic contact is crucial for the performance and long-term reliability of CNT-based integrated circuits. Specifically, the interface between A-CNTs and metal electrodes often exhibits high contact resistance, thermal instability, low injection efficiency, and so on. Palladium (Pd) has been widely used to establish ohmic contacts with CNTs in previous studies. However, under high-voltage or high-temperature conditions, the ohmic contact between Pd and CNTs may degrade due to thermal expansion coefficient (TEC) mismatches between the two materials, leading to potential device failure [11,12]. This phenomenon poses a significant challenge in integrated circuits, where stability and reliability are paramount. Consequently, there is a need to reassess the contact properties and explore alternative metal materials for A-CNTs.

Prior research has investigated the formation of stable ohmic contacts between various metals and single-walled carbon nanotubes (SWCNTs). Techniques such as high-temperature rapid annealing and ultrasonic bonding have been employed to form transition metal carbides at the metal–SWCNT interface, which reduce contact resistance ($R_{c}$) and improve contact reliability. Lee et al. reported a significant improvement in $R_{c}$ by annealing titanium (Ti) with SWCNTs, achieving reductions of several orders of magnitude [13]. Similarly, Huang and colleagues established stable ohmic contacts between CNTs of varying diameters and niobium (Nb) through annealing [14]. In recent studies, Asaka et al. investigated the effect of localized Joule heating in reducing the contact resistance between multi-walled carbon nanotubes (MWCNTs) and metals [15]. This method causes the metal to begin melting at the contact surface, allowing the carbon nanotubes to embed into the molten metal, thereby reducing contact resistance. However, precise control of current intensity is required to prevent excessive melting of the contact materials. Chai et al. successfully established low-resistance electrical contacts between various metals and individual CNTs by introducing graphene as an intermediate layer between the CNT and the metal [16]. The reduction in contact resistance was attributed to improved wettability and an increased effective contact area. Leong et al. found that in graphene—a material similar to carbon nanotubes—annealing improves metal–graphene contact performance by forming more end-contacts at the chemisorbed metal–graphene interface through the carbon dissolution–precipitation mechanism [17]. In previous studies, there has been a lack of research on the formation of ohmic contacts between A-CNTs and metals following rapid annealing processes.

In this study, we present a detailed investigation of a rapid thermal annealing (RTA) process that facilitates the formation of transition metal carbides at the interface between titanium (Ti), nickel (Ni), and A-CNTs. This process reduces both $R_{c}$ and the Schottky Barrier Height (SBH), while mitigating the impact of impurities and organic residues from the A-CNT production process. Transition metal carbides are known for their excellent electrical conductivity, high melting points, superior corrosion resistance, and low diffusion coefficients, making them ideal candidates for this application [18,19,20,21]. Using the transfer length method (TLM), we measured $R_{c}$ and conducted a comparative analysis of Ti and Ni devices before and after annealing. Our results show that the $R_{c}$ for both Ti and Ni contacts decreased by a factor of 60–70. Moreover, precise measurements revealed a significant reduction in the SBH at the Ti and Ni contacts. The annealing process established highly reliable ohmic contacts between Ti, Ni, and A-CNTs. These results provide a robust framework for further exploration of more efficient, stable, and scalable CNT-based CMOS technology, paving the way for future nanoelectronics applications.

## 2. Experiments

**Figure 1:**
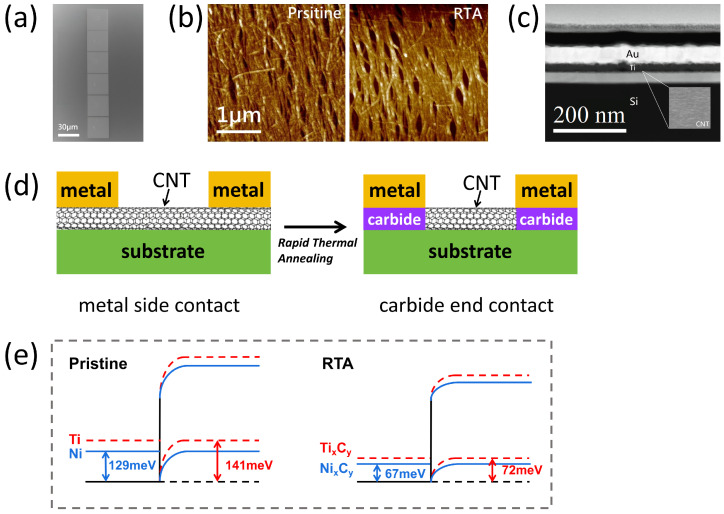
(**a**) Scanning electron microscopy(SEM) images of the fabricated CNT TLM test structures. The scale bar is 30 µm. (**b**) Atomic force microscopy (AFM) images of the A-CNT surface before and after 700 °C rapid annealing. (**c**) A cross-sectional schematic illustration of the metal–A-CNT contact interface in the TLM test device, with an inset showing a high-resolution SEM image of the A-CNTs. (**d**) Metal carbide end-bonded contact through rapid thermal annealing. (**e**) Through rapid annealing, the SBH between Ti and Ni metals and carbon nanotubes is reduced.

Aligned carbon nanotube (A-CNT) arrays, characterized by high density and purity, were fabricated on silicon substrates using the dimension-limited self-alignment (DLSA) method, as detailed in reference [22]. In this study, the linear density of the A-CNTs used was about 230 tubes/μm, and the carbon nanotubes exhibited a single-walled structure with a diameter of 1.12 nm (±0.22). We utilized the yttrium sacrificial layer process [23] and ultraviolet ozone (UVO) treatment [24], ensuring the formation of high-quality metal/A-CNT interfaces. To evaluate the impact of RTA on the reduction of $R_{c}$, TLM test structures with varying channel lengths were designed. A scanning electron microscope (SEM) image of the TLM test structure is shown in Figure 1a. The electrode regions were first precisely defined using EBL. Following this, metal stacks of 20/50 nm Ni/Au, 20/50 nm Ti/Au, and 20/50 nm Pd/Au were deposited using electron beam evaporation, followed by a lift-off process. A cross-sectional SEM image of the Ti/Au metal stack on the A-CNT films is shown in Figure 1c.

**Figure 2:**
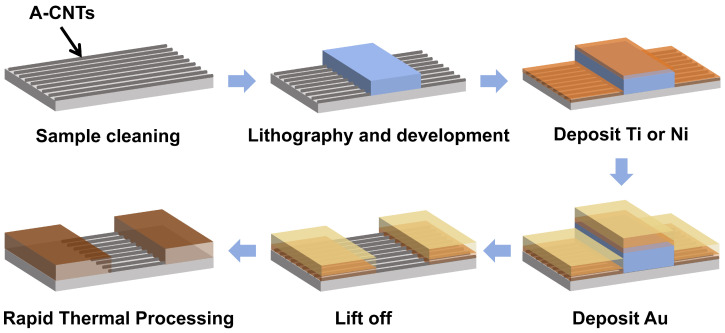
A schematic of the key steps in the fabrication of carbon-based devices using the RTA process.

The test device was annealed in a rapid thermal processing furnace under a nitrogen atmosphere with an environmental pressure flow rate of 30 Standard Liters per Minute (SLPM), at a heating rate of approximately 23 °C/s. Upon reaching the target temperature, isothermal holding was maintained for 30 s followed by natural cooling in the nitrogen environment. Figure 1d shows a schematic of the metal–CNT contact scheme used in this article. After rapid annealing, CNT and Ti form transition metal carbides. Figure 1b presents atomic force microscopy (AFM) images of the A-CNTs before and after 700 °C RTA, demonstrating that the RTA process induces negligible alterations in both the surface morphology and alignment orientation of the A-CNTs. Figure 2 illustrates the key fabrication steps for the carbon-based devices using the RTA process. After the RTA process, transition metal carbides were formed to enhance the contact strength. To characterize the effect of RTA on the contact properties, current measurements were performed both before and after annealing, and the $R_{c}$ was extracted. Furthermore, the SBH was measured before and after annealing over a temperature range of 213 K to 373 K, and the results were compared. As shown in Figure 1e, the reduction in SBH after annealing at 700 °C is presented in comparison with the pristine state.

Finally, to assess the stability of the Ti/Au and Ni/Au contacts after RTA, the devices were subjected to increasing current and voltage. Changes in the resistance were monitored to determine the conditions under which the ohmic contact would fail.

## 3. Results and Discussion

During the RTA process of Ti/A-CNT contact, oxidation plays a crucial role in controlling the solid-state reaction that forms titanium carbide (${\mathrm{Ti}}_{x}C_{y}$) at the interface between Ti and the A-CNT films. Given Ti’s high sensitivity to oxygen, under high-temperature conditions, Ti is more likely to react with $O_{2}$ to form titanium dioxide (Ti$O_{2}$) rather than forming ${\mathrm{Ti}}_{x}C_{y}$ with CNTs [25]. Consequently, it is essential to maintain a high-vacuum environment during the electron beam evaporation process and to perform the RTA in a nitrogen atmosphere to minimize unwanted oxidation. During the RTA process for the Ni/A-CNT contact, we employed the same process flow as that used for the Ti/A-CNT contact. The temperature was rapidly raised to the target annealing temperature under a nitrogen atmosphere, held for 30 s, and then allowed to cool naturally.

**Figure 3:**
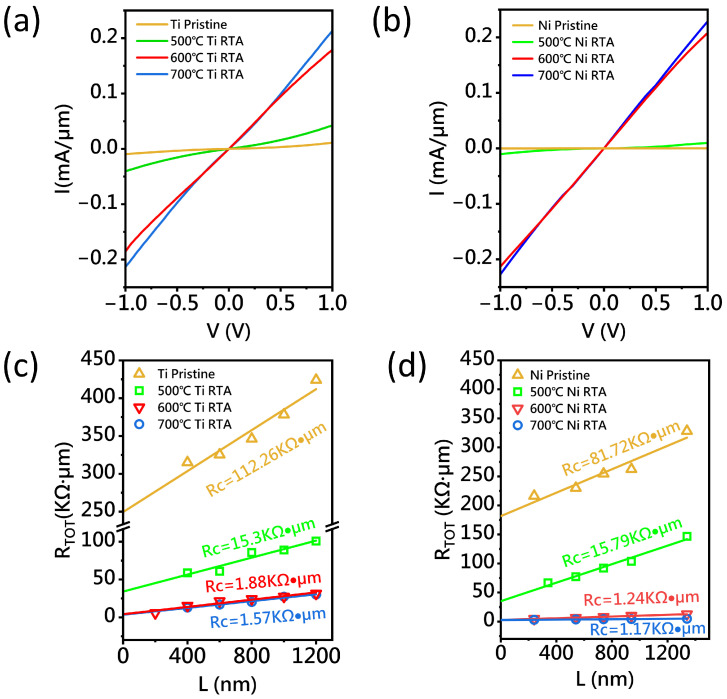
(**a**) The current–voltage (I–V) characteristics of the Ti-based devices before and after rapid thermal annealing. (**b**) The I–V characteristics of the Ni-based devices before and after rapid thermal annealing. (**c**) The average total resistance $R_{\mathrm{TOT}}$ for the TLM device after Ti contact annealing as a function of the channel length $L_{\mathrm{ch}}$: the contact resistance $R_{c}$ drops from 112.26 kΩ·μm to 1.57 kΩ·μm after annealing at 700 °C. (**d**) The average total resistance $R_{\mathrm{TOT}}$ for the TLM device after Ni contact annealing as a function of the channel length $L_{\mathrm{ch}}$: the contact resistance $R_{c}$ drops from 81.72 kΩ·μm to 1.17 kΩ·μm after annealing at 700 °C.

Figure 3a illustrates the current–voltage (I–V) characteristics of Ti/A-CNT contacts after RTA at temperatures of 500 °C, 600 °C, and 700 °C. At 500 °C, the current shows only a slight increase, indicating that the temperature is insufficient for the formation of titanium carbide. However, at 600 °C and 700 °C, the I–V characteristics become linear, suggesting the formation of typical ohmic contacts. Notably, after annealing at 700 °C, the output current increases by a factor of 22 compared to the non-annealed sample. To extract the $R_{c}$, a TLM test structure with channel lengths ranging from 200 nm to 1340 nm and a channel width of 30 μm was employed. The total resistance ($R_{\mathrm{TOT}}$) of the TLM structure can be expressed as

(1) $$R_{\mathrm{TOT}}=2R_{c}+R_{\mathrm{ch}}.$$

where $R_{\mathrm{TOT}}$ is the total resistance, $R_{\mathrm{ch}}$ is the channel resistance, and 2$R_{c}$ accounts for the resistance of the two contacts. By performing a linear fit of the TLM plot, $R_{c}$ can be extracted from the intercept of the fitted line, while the channel resistance $R_{\mathrm{sh}}$ is related to the slope of the linear fit [26,27,28]. It should be noted that while the TLM measurement is susceptible to discrepancies between the designed and actual channel lengths from the lithography process, as well as material inhomogeneity in carbon nanotubes, the present study employs single-step lithography to fabricate TLM structures with an enlarged contact width of 30 μm—significantly exceeding the diameter of individual CNTs—thereby minimizing measurement errors. Following the extraction of $R_{c}$, normalization with respect to the channel width is performed to obtain the contact resistance per unit width ($R_{c}$/μm). For the TLM test structure with a 30 μm wide channel, the original $R_{c}$ value is divided by the channel width, yielding the normalized ($R_{c}$/μm). This procedure effectively eliminates dimensional dependence in contact resistance characterization, enabling the direct comparison of lateral electrical parameters across different technology nodes. As shown in Figure 3c, the $R_{c}$ for the Ti/A-CNT contacts decreases dramatically from 112.26 kΩ·μm to 1.88 kΩ·μm and 1.57 kΩ·μm (drops by two orders of magnitude) after 600 °C and 700 °C annealing, respectively. This improvement in contact resistance can be attributed to the titanium carbide formation. To verify the reproducibility of the process, as shown in Figure 5c, repeated tests were conducted on five Ti/A-CNT contacts at 600 °C and 700 °C. The results demonstrate that the RTA process can consistently reduce $R_{c}$.

Nickel (Ni) is widely known for its good electrical conductivity and has been used extensively in the semiconductor industry. Through a solid-state reaction at an elevated temperature, Ni reacts with carbon to form nickel carbides (${\mathrm{Ni}}_{x}C_{y}$) [29]. However, to date, the ohmic contacts between ${\mathrm{Ni}}_{x}C_{y}$ and A-CNTs has not been well explored. In this study, Ni was deposited onto the A-CNT surface by electron beam evaporation, and the subsequent process steps were similar to those used for Ti/A-CNT contacts. The samples were annealed at 500 °C, 600 °C, and 700 °C. As shown in Figure 3b, after annealing at 600 °C and 700 °C, the output current increased significantly by a factor of 20 compared to the non-annealed sample. However, the 500 °C sample showed little change in current, suggesting that ${\mathrm{Ni}}_{x}C_{y}$ phase was formed insufficiently. Figure 3d shows the extracted $R_{c}$ for the annealed Ni/A-CNT samples at different temperatures. After 600 °C and 700 °C annealing, $R_{c}$ decreased from 81.72 kΩ·μm to 1.24 kΩ·μm and 1.17 kΩ·μm, respectively. These results indicate that Ni reacted with carbon to form ${\mathrm{Ni}}_{x}C_{y}$ phase, achieving good ohmic contact.

**Figure 4:**
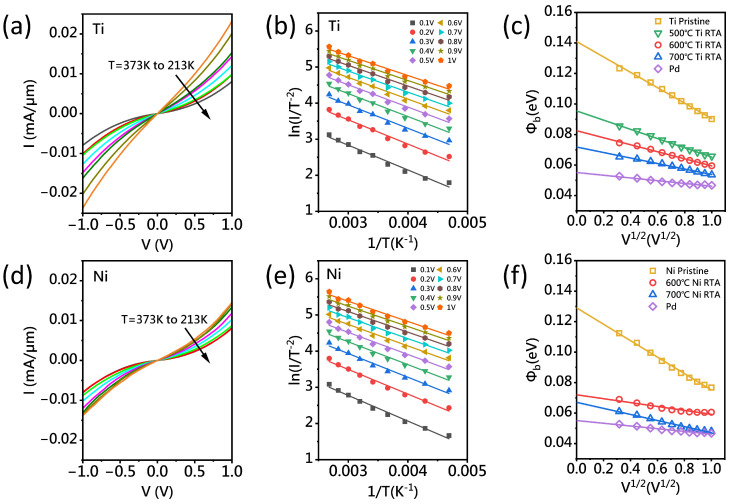
(**a**) Effective I–V characteristics of the unannealed Ti device from 213 K to 373 K. (**b**) For Ti devices, the ln($I/T^{-2}$) vs. 1/T gradient describes the SBH from 213 K to 373 K at V (0.1–1 V). (**c**) SBH as a function of the square root of voltage V ($V^{1/2}$), SBH of Ti devices at zero bias voltage after rapid thermal annealing at 500, 600, and 700 °C, respectively, and the SBH of Pd device added for comparison. (**d**) The effective I–V characteristics of the unannealed Ni device from 213 K to 373 K. (**e**) For Ni devices, the ln($I/T^{-2}$) vs. 1/T gradient describes the SBH from 213 K to 373 K at V (0.1–1 V). (**f**) The SBH of Ni devices at zero bias voltage after rapid thermal annealing at 600 and 700 °C.

Typically, transition metal on semiconducting CNTs forms a Schottky contact, where the current transport mechanism involves both thermionic emission and quantum tunneling through the metal–CNT interface barrier. As the SBH decreases, electrons and holes are able to more easily cross the barrier, leading to an increase in current, indicating that the SBH has a significant impact on the contact resistance [7]. In this study, the SBH was carefully measured using the TLM test structure with a fixed channel length of 200 nm, where quasi-ballistic transport is expected. Figure 4a,d show the I–V data collected from Ti/A-CNT and Ni/A-CNT contacts, respectively. At lower temperatures, the current intensity is lower compared to higher temperatures, suggesting a thermionic emission mechanism and confirming the presence of a Schottky barrier between the CNT and metal contacts. To assess the role of the annealing process in reducing the barrier and contact resistance, SBH measurements were taken before and after annealing, within a temperature range of 213 K to 373 K. The data were fitted to the Schottky transport model for electrons [30], as described by

(2) $$I\propto T^{2}\;\cdot \;\exp\left(-\frac{e\Phi_{e}}{kT}\right)$$

where *T* is the temperature, *k* is the Boltzmann constant, and $\Phi_{e}$ is the effective Schottky barrier height. Figure 4b,e present the Ti and Ni devices, respectively, where the ln($I/T^{-2}$) vs. 1/T gradient describes the SBH from 213 K to 373 K at V (0.1–1 V). The barrier height at V = 0 V can be estimated using [31]

(3) $$\Phi_{e}\propto \Phi_{b}-{\left(\frac{eV}{\pi \varepsilon_{i}\varepsilon_{0}}\right)}^{1/2}\;$$

where $\Phi_{b}$ is the expected barrier height, and the applied voltage (V) is varied at different test conditions.

As shown in Figure 4c, for Ti/A-CNT contacts, the SBH is approximately 141 meV before annealing, drops from to 96 meV after 500 °C annealing, further decreases to 83 meV after 600 °C annealing, and finally, turns to 72 meV after 700 °C annealing. These results indicate a significant reduction in SBH with increasing annealing temperature, which correlates with the observed decrease in $R_{c}$. As shown in Figure 4f, the SBH for Ni/A-CNT contacts presents a similar trend to that for Ti/A-CNT contacts. The SBH drops from approximately 129 meV before annealing to 72 meV and 67 meV after 600 °C and 700 °C annealing, respectively. This suggests that 700 °C-annealed Ti/A-CNT and Ni/A-CNT contacts demonstrate a nearly 50% reduction in SBH compared to the non-annealed sample. For reference, the SBH of non-annealed Pd/A-CNT contacts is 55 meV. When the applied voltage of 1 V is reached, the SBH decreases to 47 meV, which is nearly the same as that of the 700 °C-annealed Ni/A-CNT contacts.

**Figure 5:**
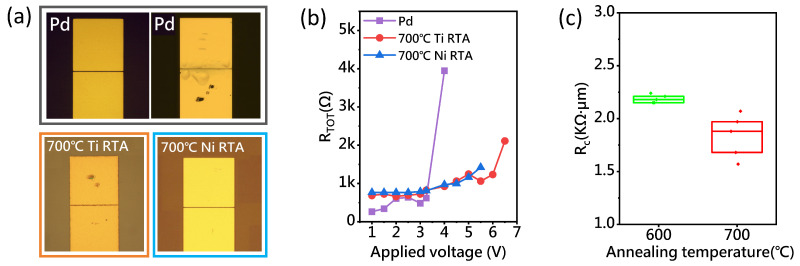
(**a**) Optical microscope images of the metal surfaces (Pd, Ti, and Ni) after ohmic contact failure testing. From top to bottom: the Pd device before testing, the Pd device after testing, the Ti device after testing, and the Ni device after testing. The surface marks on the Pd, Ti, and Ni devices were caused by probe needle tests during electrical characterization on the probe station. (**b**) The resistance from −1 V to 1 V as a function of the voltage applied to the device is shown in the figure. (**c**) The $R_{c}$ of five Ti/A-CNT contacts fabricated under identical processing conditions after RTA at 600 °C and 700 °C.

To test the stability of the fabricated ohmic contacts, the current and voltage were gradually increased, and resistance changes were monitored to determine the failure conditions. As shown in Figure 5b, in the TLM test structure with a 200 nm channel length, the voltage was varied from −1 V to 1 V, and resistance was recorded over the voltage range. In this study, we define ohmic contact failure as occurring when the resistance value exceeds 300% of the resistance measured under an applied voltage of 1 V. In addition to the resistance results, the smoothness and absence of deformation on the contact metal surface should also be taken into consideration. When the applied voltage is above 3.5 V, the resistance of Pd/A-CNT contact increases dramatically, indicating the failure of the Pd/A-CNT contact. In contrast, the Ti/A-CNT and Ni/A-CNT contacts show no significant increase in resistance, even when the applied voltage reaches 6 V, indicating that the contacts remain stable under high-voltage conditions. As shown in Figure 5a, surface morphology observations revealed that only the Pd/A-CNT contact surface exhibited deformation, while the surfaces of the Ti/A-CNT and Ni/A-CNT contacts remained largely unchanged after testing. This suggests that the poor thermal stability of the Pd/A-CNT contact is likely due to the significant difference in thermal expansion coefficients between A-CNTs and Pd [11,12], whereas the Ti/A-CNT and Ni/A-CNT contacts exhibit superior stability.

While the Ni/A-CNT and Ti/A-CNT contacts demonstrate elevated contact resistance relative to their Pd-based counterparts under high-voltage and high-temperature conditions, their interfaces with CNTs establish superior covalent–ionic hybrid bonding configurations. The initial interface formation may exhibit nanoscale interfacial discontinuities arising from incomplete carbonization processes attributed to residual organic contaminants. Crucially, voltage/thermal activation mechanisms promote progressive carbonization, effectively eliminating defect states and establishing percolation-stable conductive networks. In contrast, Pd/A-CNT interfaces predominantly rely on physisorption interactions and weak van der Waals bonds. The substantial thermal expansion coefficient (TEC) mismatch between Pd (α≈ 11.8e−6 K^−1^) and CNTs (α≈ 1e−6 K^−1^) induces thermomechanical delamination during thermal cycling, ultimately leading to contact failure. Following the formation of transition metal carbides, the robust covalent–ionic hybrid bonding between carbon nanotubes and the metal minimizes the effects of differing TECs. Notably, comparative SBH analysis at 1 V bias reveals equivalent charge injection characteristics between the Ni/A-CNT and Pd/A-CNT interfaces, suggesting that optimized Ni contacts could attain performance parity with conventional Pd contacts.

## 4. Conclusions

This study demonstrates the effectiveness of RTA in forming high-quality ohmic contacts between Ti, Ni, and A-CNTs. The annealing process facilitates the formation of transition metal carbides at the metal–CNT interface, leading to substantial reductions in both $R_{c}$ and SBH. Specifically, $R_{c}$ decreased by over an order of magnitude for the Ti/A-CNT and Ni/A-CNT contacts, reaching 1.57 kΩ·μm and 1.17 kΩ·μm, respectively, while SBH was reduced by approximately 50%, approaching the levels seen in Pd/A-CNT contacts. Furthermore, these transition metal carbide contacts exhibit superior thermal and voltage stability, outperforming Pd under harsh conditions. These findings lay the foundation for the development of more scalable, efficient, and reliable CNT-based nanoelectronics.

## Data Availability

The original contributions presented in this study are included in the article. Further inquiries can be directed to the corresponding author.
